# Machine learning-based identification of CYBB and FCAR as potential neutrophil extracellular trap-related treatment targets in sepsis

**DOI:** 10.3389/fimmu.2023.1253833

**Published:** 2023-10-13

**Authors:** GuoHua You, XueGang Zhao, JianRong Liu, Kang Yao, XiaoMeng Yi, HaiTian Chen, XuXia Wei, YiNong Huang, XingYe Yang, YunGuo Lei, ZhiPeng Lin, YuFeng He, MingMing Fan, YuLing An, TongYu Lu, HaiJin Lv, Xin Sui, HuiMin Yi

**Affiliations:** ^1^ Department of Surgical Intensive Care Unit, The Third Affiliated Hospital of Sun Yat-sen University, Guangzhou, Guangdong, China; ^2^ Guangdong Key Laboratory of Liver Disease Research, The Third Affiliated Hospital of Sun Yat-sen University, Guangzhou, China; ^3^ Key Laboratory of Liver Disease Biotherapy and Translational Medicine of Guangdong Higher Education Institutes, The Third Affiliated Hospital of Sun Yat-sen University, Guangzhou, China

**Keywords:** Sepsis, NETs, CYBB, FCAR, Sulforaphane

## Abstract

**Objective:**

Sepsis related injury has gradually become the main cause of death in non-cardiac patients in intensive care units, but the underlying pathological and physiological mechanisms remain unclear. The Third International Consensus Definitions for Sepsis and Septic Shock (SEPSIS-3) definition emphasized organ dysfunction caused by infection. Neutrophil extracellular traps (NETs) can cause inflammation and have key roles in sepsis organ failure; however, the role of NETs-related genes in sepsis is unknown. Here, we sought to identify key NETs-related genes associate with sepsis.

**Methods:**

Datasets GSE65682 and GSE145227, including data from 770 patients with sepsis and 54 healthy controls, were downloaded from the GEO database and split into training and validation sets. Differentially expressed genes (DEGs) were identified and weighted gene co-expression network analysis (WGCNA) performed. A machine learning approach was applied to identify key genes, which were used to construct functional networks. Key genes associated with diagnosis and survival of sepsis were screened out. Finally, mouse and human blood samples were collected for RT-qPCR verification and flow cytometry analysis. Multiple organs injury, apoptosis and NETs expression were measured to evaluated effects of sulforaphane (SFN).

**Results:**

Analysis of the obtained DEGs and WGCNA screened a total of 3396 genes in 3 modules, and intersection of the results of both analyses with 69 NETs-related genes, screened out seven genes (*S100A12*, *SLC22A4*, *FCAR*, *CYBB*, *PADI4*, *DNASE1*, *MMP9*) using machine learning algorithms. Of these, *CYBB* and *FCAR* were independent predictors of poor survival in patients with sepsis. Administration of SFN significantly alleviated murine lung NETs expression and injury, accompanied by whole blood CYBB mRNA level.

**Conclusion:**

CYBB and FCAR may be reliable biomarkers of survival in patients with sepsis, as well as potential targets for sepsis treatment. SFN significantly alleviated NETs-related organs injury, suggesting the therapeutic potential by targeting CYBB in the future.

## 1 Introduction

Sepsis is an immune dysfunction syndrome caused by pathogens and accompanied by severe clinical manifestations, including inflammation-related injury and multiple organ dysfunction (1). More than 18 million cases of severe sepsis occur annually, resulting in fatality rates as high as 30%–70% (2). Sepsis has gradually become the leading cause of death in non-cardiac patients in intensive care units, but the underlying pathological and physiological mechanisms remain unclear.

Neutrophils dominate the innate immune response during sepsis (3). The chemotaxis, phagocytosis, and bactericidal functions of neutrophils, which are established to occur during sepsis, are involved in the process of multiple organ failure via a complex mechanism (4, 5). Neutrophil extracellular traps (NETs) are composed of nuclear DNA, forming a scaffold structure. Antimicrobial peptides, histones, and various bactericidal factors are attached to the scaffold of nuclear DNA, NETs are function by immobilization, capture, and killing of pathogens (6). During sepsis, the activation of NETs by toll-like receptors (TLRs) plays a crucial role. However, when NETs overdevelop, the excessive release of inflammatory mediators triggers a cascade of continuous NETs release, which can result in substantial tissue and organ damage (7). Hence, NETs are an essential component of organ failure during sepsis (8), This phenomenon has garnered significant attention in the field of medical research due to its implications in the pathogenesis of sepsis-related complications. Currently, several targets related to neutrophil extracellular traps (NETs) have been identified, such as PAD4 inhibitors, TLR4 inhibitors, DNA degraders, and NET-inhibitory factors, which can reduce the release of NETs. Among them, the PAD4 inhibitor CI-Amidine, targeting PAD4, effectively inhibits the generation of NETs in septic mice and improves their survival. TAK-242 and Eritoran, as TLR4 inhibitors, have entered clinical trials but have not been able to improve the 28-day mortality rate in severe sepsis patients (9). Therefore, there is an urgent need for more NETs-related targets in sepsis to improve the survival of sepsis patients.

Weighted Gene Coexpression Network Analysis (WGCNA) can link gene clusters with highly correlated expression levels to related phenotypic traits (10), as well as categorizing genes into several modules, corresponding to clinical traits (11, 12), facilitating identification of classes of synergistic gene clusters. Moreover, least absolute shrinkage and selection operator (LASSO) regression and root mean square error (RMSE), a new type of machine learning algorithm, can more clearly define target genes. Here, we used these approaches to elucidate the mechanisms underlying NETs-mediated sepsis, providing evidence applicable to both diagnosis of severe sepsis and establishment of therapeutic strategies.

## 2 Materials and methods

### 2.1 Data download and preprocessing

Bioinformatics methods were applied to capture experimental data related to sepsis. Methods were implemented using the GEOquery software package in R (version 4.1.1, http://r-project.org/) (13). Freely available data were downloaded from the NCBI Gene Expression Omnibus (GEO)(https://www.ncbi.nlm.nih.gov/geo/), including the *Homo sapiens* sepsis-related expression profiles: GSE65682 and GSE145227. The GSE65682 dataset contains data from blood samples from 760 patients with sepsis and 42 healthy controls, while GSE145227 comprises blood sample data from 10 patients with sepsis and 12 healthy controls. Data were processed for repeat and missing values, as well as for consistency, data sorting, and outliers. Finally, the resulting clinical information was used for bioinformatic analysis. Genes related to NETs (n = 69) were derived from a report by Zhang et al. (14).

Normal and tumor tissue expression data were obtained from the GTEx database and The Cancer Genome Atlas (TCGA), which provides RNA-sequencing and clinical data for 33 types of cancer.

### 2.2 WGCNA

The first step in WGCNA was to select a soft threshold, which is a method of deriving an optimal limit value. The formula applied was: ${\hat{x}}_{i}=\text{soft(}z_{i}{\text{λ}w}_{i})=sign(z_{i})max\{0,|z_{i}|-{\text{λ}w}_{i}\}$ .

The scale-free topology fitting index was 1 to 20. Using a continuous adjacency matrix, the constructed network was fit to the power law distribution, which was closer to the actual data. Then, topology overlap and adjacency matrices (minModuleSize = 30 for module splitting) were constructed according to the gene expression data. Using a flexible dynamic tree cutting algorithm, dendrograms were generated including identifiable modules in different colors. Finally, modules significantly related to phenotype data were identified, and module eigengenes determined for each module.

### 2.3 Identification of differentially expressed genes associated with NETs

WGCNA-derived module genes, DEGs, and NETs-related genes were intersected, machine learning used to narrow the range, and then ROC curves drawn for screened NETs-related genes classified into high- and low-expression groups. Clinical data from GSE65682 were used to draw Kaplan-Meier (KM) curves to further clarify molecules associated with sepsis diagnosis and patient survival.

### 2.4 Machine learning

The LASSO method is a compression estimation approach based on the idea of reducing the variable set. By constructing a penalty function, it can compress the coefficients of variables, so that some regression coefficients become 0, to achieve variable selection.

As a binary classification model, support vector machines (SVMs) map the feature vectors of instances to points in space and determine the best classification by dividing the hyperplane with the largest interval. Through further screening of the 13 genes identified by intersection analysis, the point with the smallest cross-validation error was marked, to screen out target genes.

### 2.5 Single gene expression and organ distribution

The Human Protein Atlas (HPA) database (version: 21.1) (https://www.proteinatlas.org/) was used to construct mRNA expression maps of seven core NETs-related genes.

### 2.6 DEG screening and functional enrichment analysis

To identify DEGs between sepsis and healthy groups, the limma package in R was used to screen for differential genes (threshold, adjusted P (adjP) < 0.05, log_2_ fold-change (FC) > |0|). Further, the ggplot2 package was used to generate volcano plots, heatmaps, and Venn diagrams.

Gene ontology (GO) enrichment analysis was applied to predict gene function. GO classifies gene functions into three categories: cellular component (CC), molecular function (MF), and biological process (BP). Kyoto Encyclopedia of Genes and Genomes (KEGG) is a widely-used database that stores information about genomes, biological pathways, diseases, and drugs. GO and KEGG enrichment analyses were performed and visualized using the GOplot package, with false discovery rate < 0.05 considered statistically significant. Pathview (15) was used to visualize KEGG pathways associated with NETs.

### 2.7 Protein-protein interaction network construction

The STRING database (http://string-db.org, version 11.5) online tool was used to predict and visualize PPI network models based on the seven screened NETs-related genes.

### 2.8 Prediction of related microRNAs, transcription factors and drugs

Regnetwork (https://regnetworkweb.org/) (16) was used to predict miRNAs and TFs upstream of the seven screened genes, and Cytoscape software (17) applied to construct and visualize the central gene-TF-miRNA interaction network. NetworkAnalyst (18)(https://www.networkanalyst.ca/) was applied to analyze the interactions between hub genes and drugs, including chemical and gene interactions.

### 2.9 Gene set enrichment analysis

GSEA is a method for assessing whether a gene set differs significantly between two biological states. Expression datasets are often used to estimate changes in pathways and biological processes. GSEA was performed using gene sets downloaded from the MSigDB database (https://www.gsea-msigdb.org/gsea/msigdb/index.jsp); P < 0.05 was considered statistically significant.

### 2.10 Immune cell infiltration analysis

CIBERSORT (19) is an algorithm that uses gene-based deconvolution to infer 23 human immune cell types, and applies marker gene signatures to quantify the relative scores for each cell type. To enhance the robustness of the results, CIBERSOFT uses Monte Carlo sampling to obtain deconvolution P values for each sample, and the abundance of immune cells can also be estimated based on gene expression data. Here, gene expression matrix data were uploaded to CIBERSORT and differences with *P* < 0.05 considered significant. Bar charts were generated in R using ggplot2. In addition, ggboxplot (20) was used to draw boxplots to visualize the correlation of infiltration of 23 immune cell types. Furthermore, ggplot2 was used to visualize the relationship between sepsis-related NETs and immune cells.

### 2.11 Clinical correlation analysis

To investigate the clinical prognostic value of NETs-related genes in sepsis, receiver operating characteristic (ROC) curves and KM curves were used to screen meaningful genes, and a nomogram, including multiple clinical features, established to predict the 7-, 14-, 21-, and 28-day survival of patients with sepsis. Nomogram accuracy was evaluated using calibration curve analysis.

### 2.12 Animal experiments

Mice (male; body weight, 25–30 g,C57/BL6) were obtained from the Animal Research Center of Nanjing University (Nanjing). Animals were housed and maintained in a pathogen-free facility at Sun Yat-sen University Experimental Animal Center, according to the Sun Yat-sen University “*Guidelines for Experimental Animals*”. Mice were provided with standard laboratory water and food and maintained at 20°C and 50% humidity, with a 12 h light/dark cycle.

A cecal ligation and puncture (CLP) mouse sepsis model was generated, and mice were sacrifacied after 2 days. To construct the CLP model, mice were anesthetized by intraperitoneal injection with sodium pentobarbital (50 mg/kg) until there was no response to pinching of the toes using forceps. The abdominal skin was prepared using depilatory ointment, disinfected with iodine three times, and a sterile towel used to cover the surgical field. Then, a 1.5–2 cm midline incision of the abdomen was made to expose the cecum. In the sham operation group, the was cecum exposed but not treated. In the CLP group, the cecum was ligated with 6-0 non-absorbable sterile surgical silk at a distance halfway between the distal end and the base of the cecum. Next, the cecum was punctured 1 cm from the distal end using a 21G sterile needle; before cecal perforation, the cecum contents were pushed to the distal end of the cecum, and puncture of blood vessels was avoided. After removing the needle, a small amount of feces was squeezed from the puncture hole, to ensure patency. Finally, the wound was sutured layer-by-layer. Postoperative fluid rehydration was conducted by subcutaneous injection of 1 ml pre-warmed 0.9% sterile saline (37°C) per mouse. For postoperative analgesia, buprenorphine (0.05 mg per kg) was injected subcutaneously every 6 h for 2 days.

### 2.13 Total RNA extraction and real-time quantitative polymerase chain reaction

Total RNA samples were extracted from whole blood for use in RT-qPCR using TRIzol (Invitrogen, Life Technologies, USA), according to the manufacturer’s instructions. Total RNA purity and concentration were evaluated using an ultraviolet spectrophotometer (BioMate 3S UV-visible spectrophotometer; ThermoFisher Scientific). Total RNA samples aliquots (1 µg) were used to synthesize cDNA, using a cDNA Synthesis Kit (Roche Applied Science, Indianapolis, USA), and qRT–PCR conducted with a reverse transcription system (LC-480, Roche, USA) using SYBR Master Mix (Roche Applied Science). The housekeeping genes, *GAPDH* and *HPRT*, served as internal controls for cDNA normalization. All primer sequences used in this study are listed in **Table S3**.

### 2.14 Immunofluorescence assay

Lung tissue specimens were cut into 4 µm sections after embedding in paraffin. Frozen tissue samples were dehydrated with 15% and 30% sucrose. Slides were deparaffinized and rehydrated using xylene and gradient ethanol concentrations (100%, 95%, 80%, 70%, and 50%). Antigen retrieval was conducted by microwaving samples in 1× EDTA buffer (pH 6.0) for 15 min. Following blocking with 1% goat serum in PBS and 0.1% Triton X-100 for 30 min, cit-H3 (CST, 83506, 1:400), Ly6G(Servicebio,GB11229,1:400) antibodies were added to slides and incubated at 4°C overnight. Slides were then washed with PBS three times and incubated with FITC-conjugated secondary antibody at room temperature for 1 h, followed by staining with DAPI for 5 min. Slides were then examined under a microscope (Zeiss).

### 2.15 Flow cytometry analysis

A total of 2 × 10^5^ bronchoalveolar lavage fluid (BALF) cells were collected and suspended in 500 μl PBS. Antibodies against Ly6G, F480, and CD45were added to cells, which were then incubated for 30 min at 4°C in the dark. After washing with 1 ml 1× PBS, sample fluorescence was analyzed using a thirteen-color FACS Calibur instrument (Beckman Coulter, Hercules, CA, USA) and FlowJo software (BD Biosciences, USA).

### 2.16 Clinical data and specimen collection

The Ethics Committee of the Third Affiliated Hospital of Sun Yat-Sen University (Guangzhou, China) approved this study, and this study was conducted in accordance with the “*Declaration of Helsinki*”, with informed consent from the patients. Three patients with sepsis after liver or kidney organ transplantation were included in the study, as well as three healthy individuals. Patient data were collected retrospectively from clinical records.

### 2.17 Statistical analysis

All data processing and statistical analyses were conducted using R software (version 4.1.1). Comparisons of continuous variables were performed using t-tests, and comparisons of categorical variables were by chi-squared analyses or Fisher’s exact test. All calculations were two-tailed and P < 0.05 was considered statistically significant.

## 3 Results

### 3.1 Identification of DEGs in training set data

**Figure 1:**
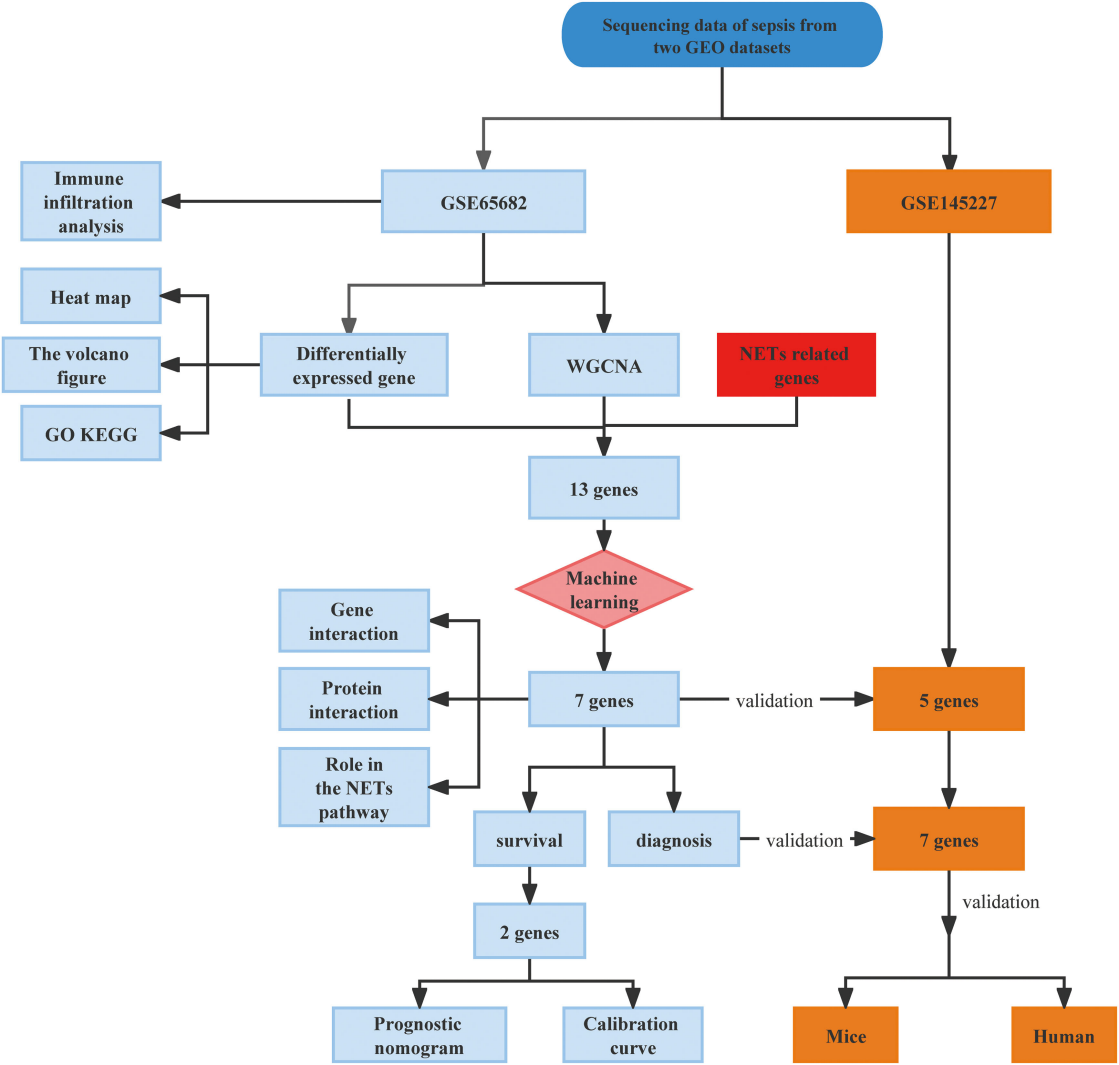
Flowchart of the study in this research.

**Figure 2:**
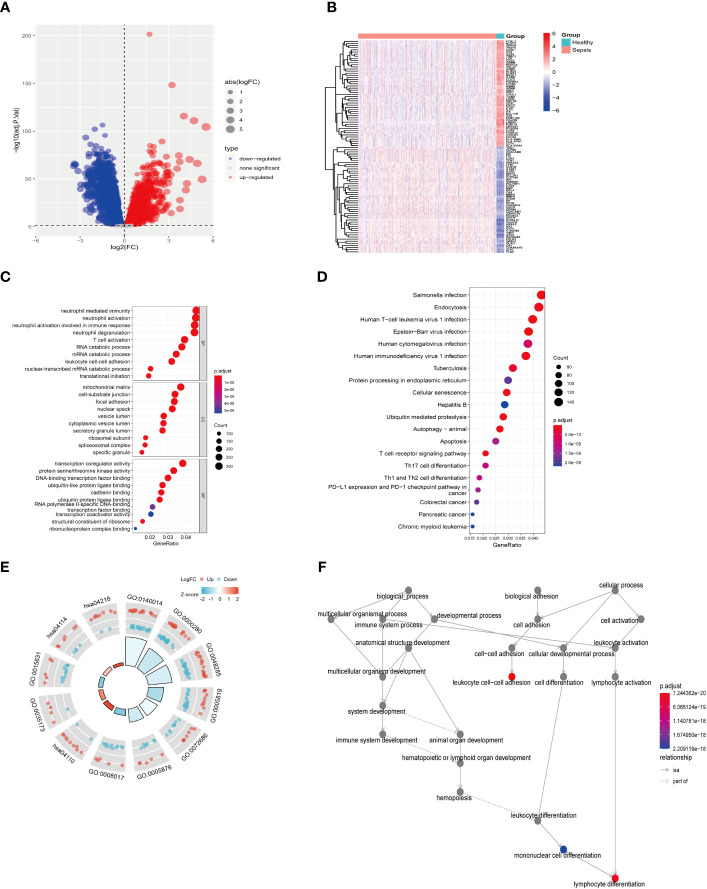
Difference analysis of training set GSE65682. **(A)** Differential analysis volcano map. **(B)** Differential analysis heat map. **(C)** GO functional enrichment analysis (including BP MF CC). **(D)** KEGG pathway enrichment analysis results. **(E)** GO KEGG circle chart analysis results. **(F)** BP topology.

First, as shown in the flowchart (**Figure 1**), we identified DEGs between the sepsis and healthy groups in the GSE65682 dataset for use in further analysis using the limma package in R. First, data were normalized (**Figures S1A, B**), and a threshold of adjP < 0.05, log_2_ FC > |0| used to screen DEGs between the healthy and the sepsis groups. A total of 8973 DEGs were obtained, including 4931 with log_2_ FC > 0 and 4041 with log_2_ FC < 0; corresponding volcano and heat maps are presented in **Figures 2A, B**. Next, GO and KEGG enrichment analysis of DEGs was conducted (**Figures 2C–E**; **Table 1**). Enriched MF categories included: neutrophil-mediated immunity, neutrophil activation, neutrophil activation involved in immune response, and neutrophil degranulation, indicating that the molecular function of sepsis is associated with neutrophils. The main enriched CC category was mitochondrial matrix. We previously demonstrated that mesenchymal stem cell exosomes can be delivered through mitochondria to treat NETs due to hepatic ischemia-reperfusion. KEGG analysis mainly identified enrichment of signaling pathways related to infection (**Figures S1C, D**). Further, topological mapping revealed that leukocyte cell-cell adhesion and lymphocyte differentiation were involved in the final biological pathway (**Figure 2F**). These data suggest that NETs play a pivotal role in sepsis.

**Table 1:** GO and KEGG pathway enrichment analysis of candidate genes in the most significant terms.

ID	Description	GeneRatio	BgRatio	pvalue	p.adjust	qvalue	zscore
GO:0140014	mitotic nuclear division	32/196	264/18670	7.53e-25	2.33e-21	2.01e-21	-0.353553391
GO:0000280	nuclear division	34/196	407/18670	4.63e-21	7.16e-18	6.16e-18	-0.685994341
GO:0048285	organelle fission	35/196	449/18670	1.10e-20	1.14e-17	9.78e-18	-0.507092553
GO:0005819	spindle	25/202	347/19717	2.04e-14	6.25e-12	5.57e-12	-1
GO:0072686	mitotic spindle	15/202	109/19717	3.72e-13	5.71e-11	5.09e-11	-0.25819889
GO:0005876	spindle microtubule	12/202	59/19717	7.07e-13	7.24e-11	6.45e-11	-0.577350269
GO:0008017	microtubule binding	16/191	246/17697	1.08e-08	4.80e-06	4.31e-06	-1.5
GO:0035173	histone kinase activity	5/191	17/17697	7.74e-07	9.93e-05	8.92e-05	1.341640786
GO:0015631	tubulin binding	16/191	336/17697	7.79e-07	9.93e-05	8.92e-05	-1.5
hsa04110	Cell cycle	11/95	124/8076	1.88e-07	3.81e-05	3.62e-05	2.110579412
hsa04114	Oocyte meiosis	10/95	129/8076	2.44e-06	2.48e-04	2.35e-04	0.632455532
hsa04218	Cellular senescence	10/95	156/8076	1.34e-05	9.06e-04	8.60e-04	1.897366596

### 3.2 WGCNA

**Figure 3:**
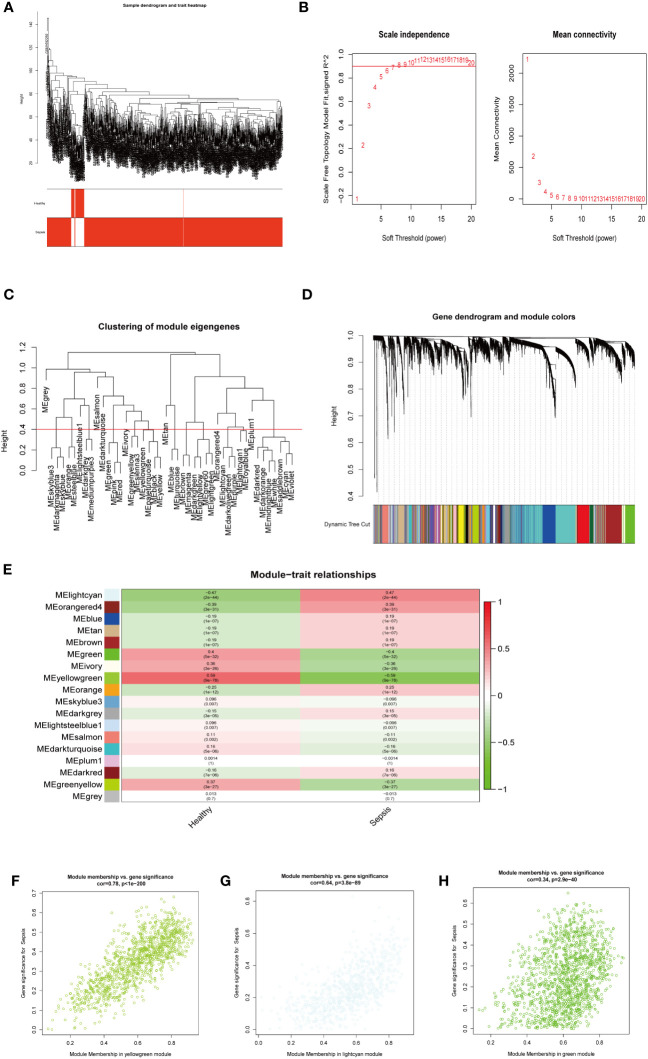
WGCNA analysis on the GSE65682 dataset. **(A)** The sample clustering dendrogram corresponding to healthy and sepsis samples. **(B)** Filter the soft threshold powers value, the most appropriate when the screening result is 6. **(C)** Clustered dendrograms are cut at a height of 0.4 to detect and combine similar modules. **(D)** Display and merge of clustering trees **(E)** Association of gene modules with clinical traits (Screen the modules whose correlation is greater than or equal to 0.4, and screen out 3 modules in total Green, lightcyan, yellowgreen). **(F)** Correlation between yellow green module genes and Sepsis traits **(G)** Correlation between light cyan module genes and Sepsis traits. **(H)** Correlation between green module genes and Sepsis traits.

Next, we investigated potential crucial gene modules associated with sepsis by using WGCNA to correlate each module with corresponding clinical features for analysis of biological processes. All genes in the GSE65682 dataset were included in WGCNA (**Figure 3A**) and screened for a suitable soft threshold powers value. The results showed that the most suitable number was 6 (**Figures 3A, B**). Subsequently, we generated and merged a clustering tree (**Figures 3C, D**), and then assessed the correlations between the modules, and found that the results failed to reach statistical significance (**Figure S2B**). Correlation analysis within modules demonstrated the reliability of the module description, while there were no substantial connections between modules (**Figure S2A**). As shown in **Figure 3E**, a total of three modules (green, light cyan, and yellow-green) with correlation co-efficient values ≥ 0.4 were screened (**Figure 3E**).

We next performed GO and KEGG enrichment analyses on the genes in the three modules identified as clinically relevant to sepsis (**Figures 3F–H**). Our results indicate that the yellow-green module genes were related to ribonucleoprotein complex biogenesis (**Figure S2C**), green module gene was related to ATPase activity (**Figure S2E**), and genes in the light cyan module were related to neutrophil degranulation, neutrophil activation involved in immune response, and neutrophil-mediated immunity, among other processes (**Figure S2D**).

Together, these results shown that three modules (green, light cyan, and yellow-green) were related to sepsis, where the light cyan module was associated with neutrophil immune processes, the yellow-green module with ribosomes, and the green module with energy activity.

### 3.3 Screening and expression of NETs-related genes in sepsis

**Figure 4:**
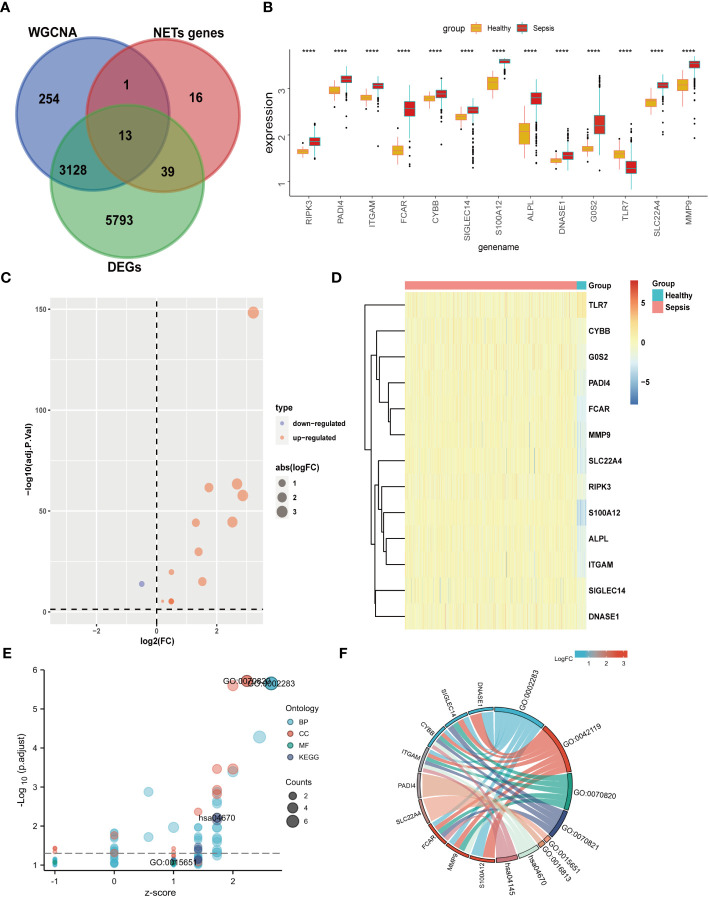
Differential expression and GO KEGG analysis of NETs- related genes in sepsis. **(A)** The genes screened by WGCNA, the difference genes are expected to take the intersection of NETs genes, and 13 genes are obtained. **(B)** Differential expression of 13 genes. **(C)** 13 genes are displayed individually with a volcano map. **(D)** 13 genes are displayed individually with a heat map. **(E)** GO KEGG bubble plot of 13 genes. **(F)** Chord diagram display of 13 genes. (****p<0.0001).

Our data from both DEGs and WCNA indicated that biological processes involving neutrophils have an important impact on sepsis, and NETs are established to have critical roles in sepsis. Therefore, we speculated that key NETs-related genes are likely to have important functions in sepsis. Based on a literature search, we obtained 69 NETs-related genes. By intersecting data from WGCNA (3396 genes) and DEGs (8973 genes) with the 69 NETs-related genes, we identified 13 genes (**Figure 4A**): *RIPK3*, *PADI4*, *ITGAM*, *FCAR*, *CYBB*, *SIGLEC14*, *S100A12*, *ALPL*, *DNASE1*, *G0S2*, *TLR7*, *SLC22A4*, and *MMP9*. These 13 genes were then compared with the GSE65682 dataset, and they were found to be significant DEGs between the healthy and sepsis groups (**Figures 4B–D**).

GO KEGG enrichment analysis of these 13 genes was then performed using the clusterprofiler package in R (**Figures 4E**, **S3**; **Table 2**), and the identified enriched pathways were closely related to NETs. We also analyzed the relationship between each gene and pathway (**Figure 4F**; **Table 3**) and the resulting data indicated that most genes were related to neutrophil activation.

**Table 2:** GO and KEGG enrichment analysis for NETs related genes.

ID	Description	GeneRatio	BgRatio	pvalue	p.adjust	qvalue
GO:0002283	neutrophil activation involved in immune response	7/13	488/18670	1.20e-08	2.22e-06	1.33e-06
GO:0070820	tertiary granule	5/13	164/19717	4.57e-08	1.92e-06	1.11e-06
GO:0015651	quaternary ammonium group transmembrane transporter activity	1/13	10/17697	0.007	0.085	0.056
hsa04670	Leukocyte transendothelial migration	3/8	114/8076	1.46e-04	0.006	0.005

DEG, differentially expressed gene; GO, Gene Ontology; KEGG, Kyoto Encyclopedia of Genes and Genomes.

**Table 3:** Enrichment analysis for NETs related genes.

terms	ID	Description	GeneRatio	pvalue	geneID	Count
BP	GO:0002283	neutrophil activation involved in immune response	7/13	1.20e-08	ITGAM/FCAR/CYBB/SIGLEC14/S100A12/DNASE1/MMP9	7
BP	GO:0042119	neutrophil activation	7/13	1.38e-08	ITGAM/FCAR/CYBB/SIGLEC14/S100A12/DNASE1/MMP9	7
CC	GO:0070820	tertiary granule	5/13	4.57e-08	ITGAM/FCAR/CYBB/SIGLEC14/MMP9	5
CC	GO:0070821	tertiary granule membrane	4/13	1.21e-07	ITGAM/FCAR/CYBB/SIGLEC14	4
MF	GO:0015651	quaternary ammonium group transmembrane transporter activity	1/13	0.007	SLC22A4	1
MF	GO:0016813	hydrolase activity, acting on carbon-nitrogen (but not peptide) bonds, in linear amidines	1/13	0.008	PADI4	1
KEGG	hsa04670	Leukocyte transendothelial migration	3/8	1.46e-04	ITGAM/CYBB/MMP9	3
KEGG	hsa04145	Phagosome	3/8	3.42e-04	ITGAM/FCAR/CYBB	3

NETs.

Enrichment analysis of these 13 NETs-related genes indicated that they were also related to the NF-κB pathway and the NADPH oxidase complex (**Figure S3**). Indeed, recent studies have confirmed that NETs can function through the NF-κB pathway in both inflammation and cancer (21). Similarly, the NADPH oxidase complex can lead to the generation of reactive oxygen species (ROS), which play a crucial role in ROS-dependent NETs (22). Based on our results, the NF-κB pathway and NADPH are likely to play important roles in the involvement of NETs in sepsis.

### 3.4 Machine learning

**Figure 5:**
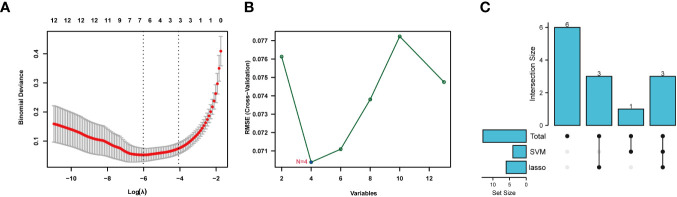
Machine learning for screening key genes. **(A)** Lasso screened 6 genes. **(B)** SVM screened 4 genes. **(C)** Get 7 genes after taking the union.

Lasso and SVM machine learning methods were used to further narrow the scope of NETs-related genes involved in sepsis. The 13 identified genes were screened by the Lasso method, which resulted in narrowing to six genes (**Figure 5A**). Further, four genes were screened by the SVM method (**Figure 5B**). Finally, seven genes were obtained after combination of the genes identified by each method: *S100A12*, *SLC22A4*, *FCAR*, *CYBB*, *PADI4*, *DNASE1*, and *MMP9* (**Figure 5C**). These seven NETs-related genes were expressed differently in normal tissues (**Figure S7**); six of the genes, except *DNASE*, were all expressed at the highest level in bone marrow. Further, levels of *FCAR* expression were second-highest in lung tissue, while those of *CYBB*, *FCAR*, *MMP9*, and *PADI4* were second-highest in lymphoid tissue.

### 3.5 Functional network construction

#### 3.5.1 Gene-gene and protein-protein correlations

**Figure 6:**
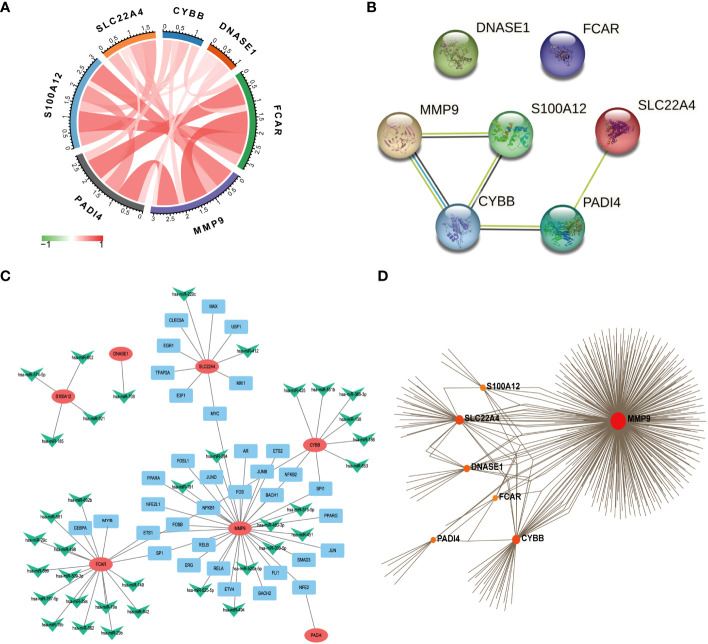
Functional network construction. **(A)** Correlation analysis of 7 core genes was performed. (The red line represents positive correlation, the green line represents negative correlation, the darker the color, the stronger the correlation). **(B)** Protein correlation analysis of 7 core genes using string database. **(C)** Use Regnetwork (https://regnetworkweb.org/) to predict miRNAs and transcription factors (TFs) upstream of 7 genes, and use cytoscape software for visualization. **(D)** Use NetworkAnalyst (https://www.networkanalyst.ca/) to analyze the relationship between hub gene and drug.

We used the R package, circlize, to conduct correlation analysis of the seven core NETs-related genes, and found that their levels were all positively correlated with one another (**Figure 6A**), indicating that these seven core genes promote each other and function together in the formation of NETs during sepsis.

**Figure 7:**
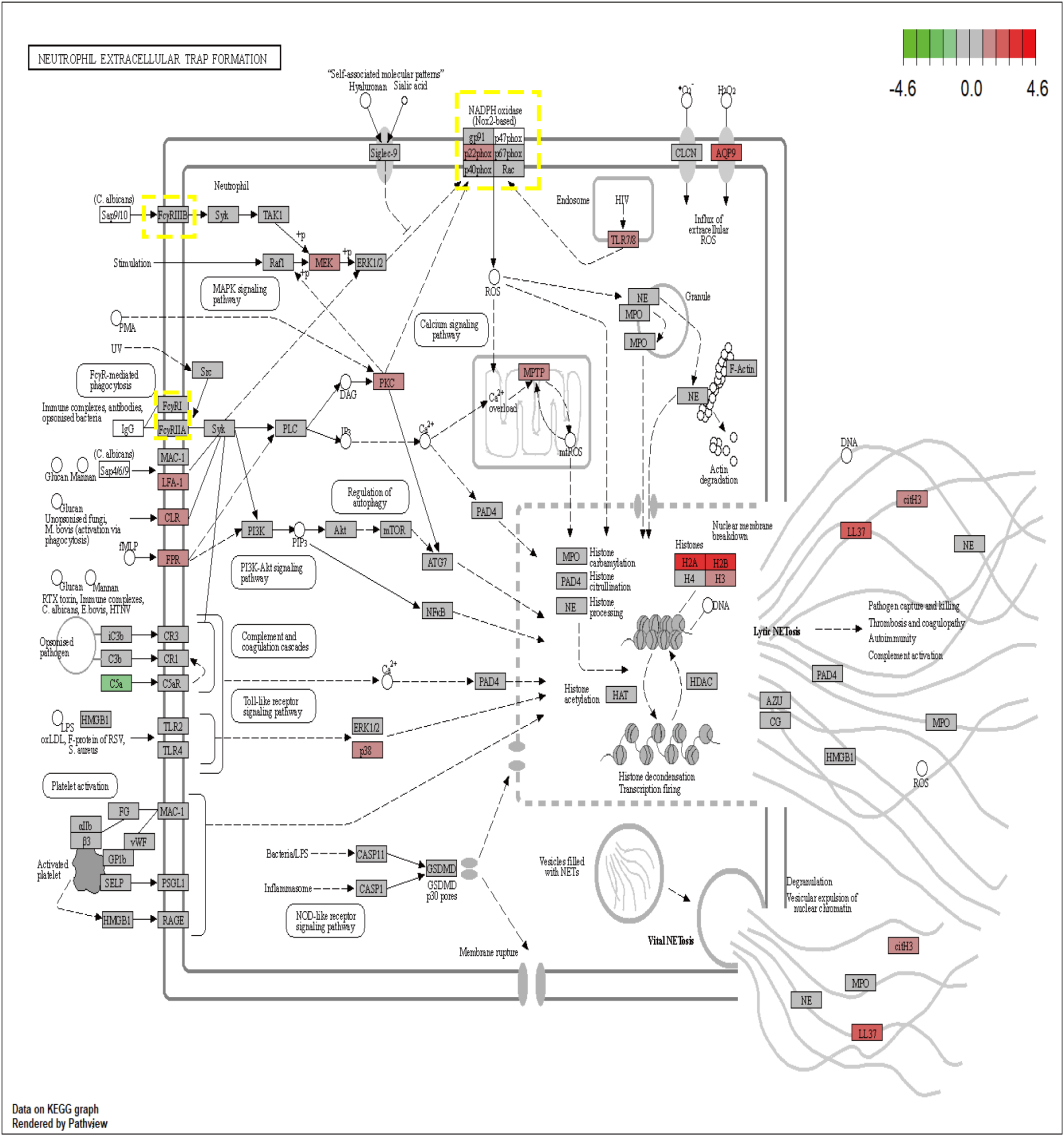
The expression changes of 7 core genes in the KEGG NETs pathway (red means increased, green means decreased).

The proteins encoded by the seven core genes were also associated with one another; DNASE1 and FCAR were independent, while the other proteins were all associated (**Figure 6B**). CitH3 and LL37 had been identified as markers of neutrophil extracellular traps (NETs). Analysis of expression changes in NETs-related KEGG pathways indicated that citH3 and LL37 tended to increase (**Figure 7**).

To clarify the respective roles of the seven NETs-related genes, correlation analysis of the seven genes with all other genes was conducted using GSE65682 data (**Figure S6**). Additionally, based on the results of the correlation analysis, single-gene GSEA was performed on reactomes using the R language clusterprofiler package. The results showed that *CYBB*, *FCAR*, *MMP9*, and *PADI4* were related to neutrophil degranulation, while *S100A12* and *SCL22A4* were associated with NETs transcription processes, and *DNASE1* was related to extracellular matrix degradation. Hence, the seven genes play different roles in different stages of NETs development in sepsis.

#### 3.5.2 MiRNAs, TFs, and drugs

We next predicted miRNAs and TFs that may be involved in regulation of the seven key genes associated with NETs using Regnetwork (https://regnetworkweb.org/) to predict miRNAs and TFs, and used Cytoscape software to visualize the results. We identified 36 miRNAs and 43 TFs in total (**Figure 6C**), among which 14 miRNAs were predicted to regulate *FCAR* had and 26 TFs were associated with MMP9; one TF was predicted to regulate multiple genes (**Table S2**); NFKB1 can regulate the three genes, *MMP9*, *FCAR*, and *CYBB*. To predict drugs that affect the seven key NETs-related genes, we used NetworkAnalyst (https://www.networkanalyst.ca/) to analyze the relationships between hub genes and drugs (**Figure 6D**); the results are presented in **Table S1**.

### 3.6 Relationship between the seven core genes and immune infiltration

**Figure 8:**
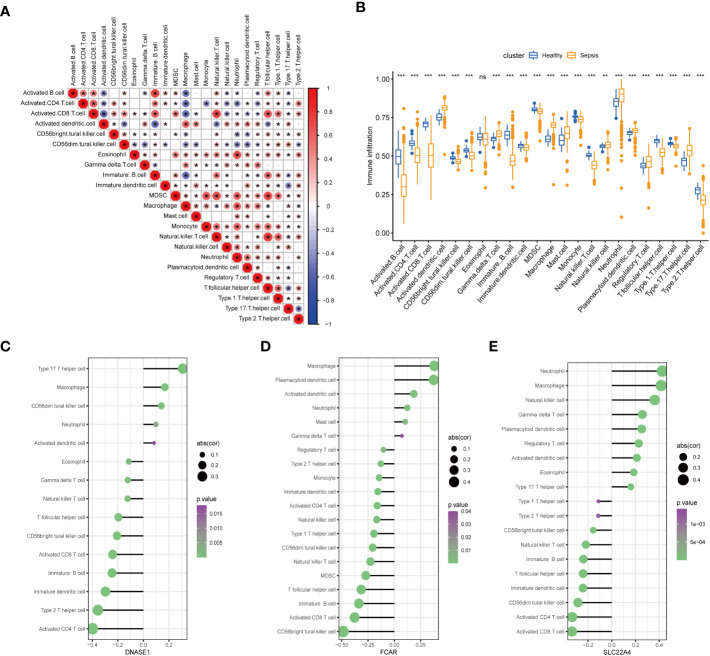
Assessment of the extent of immune cell infiltration using data from GSE65682. **(A)** Correlation between sepsis and immune cell infiltration. **(B)** Differences in immune cell infiltration between sepsis and healthy groups. **(C–E)**. Display of individual genes and immune infiltration. (*p<0.05, **p<0.01, and ***p<0.001 and ns means not significant).

To further confirm the role played by immune cells in sepsis, we evaluated the degree of immune cell infiltration using data from GSE65682 and the ssGSEA function of the GSVA package in R. Further, we analyzed the correlations between sepsis and infiltration of 23 types of immune cell (**Figures 8A, B**). According to the immune infiltration levels in the two groups (patients with sepsis and healthy controls), activated B cells, activated CD4 T cells, activated CD8 T cells, CD56-bright natural killer cells, CD56-dim natural killer cells, immature B cells, immature dendritic cells, myeloid-derived suppressor cells, monocyte natural killer T cells, T follicular helper cells, type 1 T helper cells, and type 2 T helper cells were down-regulated in the sepsis group. Further, activated dendritic cells, gamma delta T cells, macrophages, mast cells, natural killer cells, neutrophil, plasmacytoid dendritic cells, regulatory T cells, type 17 T helper cells were significantly up-regulated in sepsis; however, there was no significant difference in eosinophils between the two groups. These results suggest that immune infiltration has an important role in sepsis. Further, consistent with our results, neutrophils were significantly upregulated in sepsis.

After in-depth analysis, we found that *DNASE1*, *FCAR*, and *SLC22A4* played important roles in neutrophil infiltration (**Figures 8C–E**). Interestingly, not all of the identified seven core genes were involved in neutrophil infiltration, indicating that the process of NETs generation is not completely consistent with the neutrophil infiltration process, and warranting further exploration.

### 3.7 Analysis of the validation dataset

GSE145227 was selected as the verification set for validation analysis. First, we normalized the data (**Figures S5A, B**), and screened for DEGs using a threshold of adjP < 0.05. A total of 8868 DEGs between the two groups were obtained, among which, 3784 had log_2_ FC > 0 and 5084 log_2_ FC < 0; the corresponding volcano plot and heatmap are shown in **Figures S4A, B**. Verification analysis of the seven core genes in GSE145227 demonstrated that the expression of *MMP9*, *SLC22A4*, *FCAR*, *PADI4*, and *S100A12* differed significantly between the sepsis and healthy groups, and all of them showed an increasing trend; however, levels of *CYBB* and *DNASE1* did not differ significantly between the groups. Nevertheless, the mean level of *CYBB* expression in the sepsis group was higher than that in the healthy group (**Figure S4E**). A volcano plot and heatmap of the five DEGs are presented in **Figures S4C, D**. GSEA indicated that sepsis was primarily related to innate immunity and neutrophil degranulation (**Figure S5C**), consistent with our previous analysis and suggesting that neutrophils have an important role in sepsis.

### 3.8 Search for clinically significant genes

**Figure 9:**
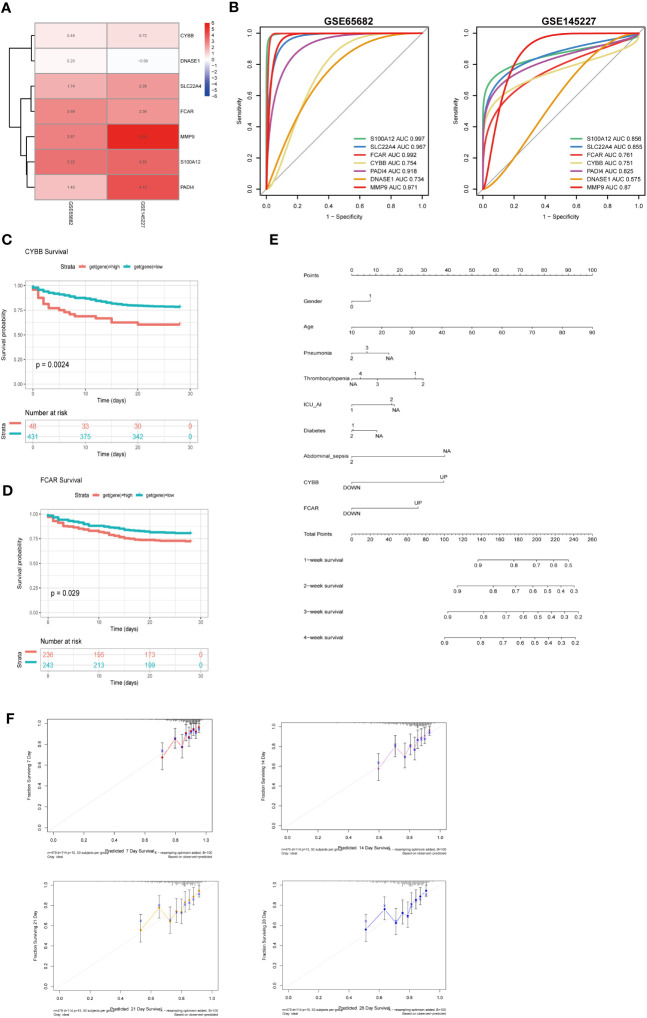
The roles of CYBB and FCAR in the diagnosis and prognosis of sepsis. **(A)** Use a heatmap to display the differential expression of 7 genes in two data sets (the value represents the logFC value of the differential analysis). **(B)** 7 genes predict the ROC curve of Sepsis (GSE65682 and GSE145227). **(C)** Survival analysis of CYBB in GSE65682 (only those with high expression and poor prognosis, p less than 0.05). **(D)** Survival analysis of FCAR in GSE65682. **(E)** Multivariate nomogram analysis **(F)** 7,14,21,28-day survival rate calibration curve.

To clarify the significance of the seven key genes related to NETs in the diagnosis of sepsis, we performed ROC analysis on the two datasets. First, we used a heatmap to visualize the DEGs in the two datasets (**Figure 9A**). In GSE66582, the genes with the top three area under the ROC curve (AUC) values were *S100A12* (0.997), *FCAR* (0.992), and *MMP9* (0.971), while in the GSE145227 dataset the genes with the top three AUC values were *MMP9* (0.87), *S100A12* (0.856), and *SLC22A4* (0.855) (**Figure 9B**).

To evaluate the significance of the 7 key NETs-related genes to patient survival, we analyzed patient data from GSE65682. Our findings implied that higher *CYBB* and *FCAR* expression were significantly and positively associated with higher mortality rates in the sepsis group (**Figures 9C, D**).

To examine whether the survival-associated genes, *CYBB* and *FCAR*, have value for prediction of clinical features in sepsis, we constructed survival prediction models including patient sex, age, pneumonia, thrombocytopenia, ICU-acquired pneumonia, diabetes, and abdominal sepsis, as well as *CYBB* and *FCAR* expression levels; these multiple factors were combined to predict the survival of patients with sepsis in ICU. Using a calibration curve fitted to patient 7-, 14-, 21-, and 28-day survival, we found that the data were evenly arranged on the diagonal, indicating that the nomogram had predictive value (**Figures 9E, F**).

### 3.9 CYBB and FCAR are critical targets for treatment of sepsis

**Figure 10:**
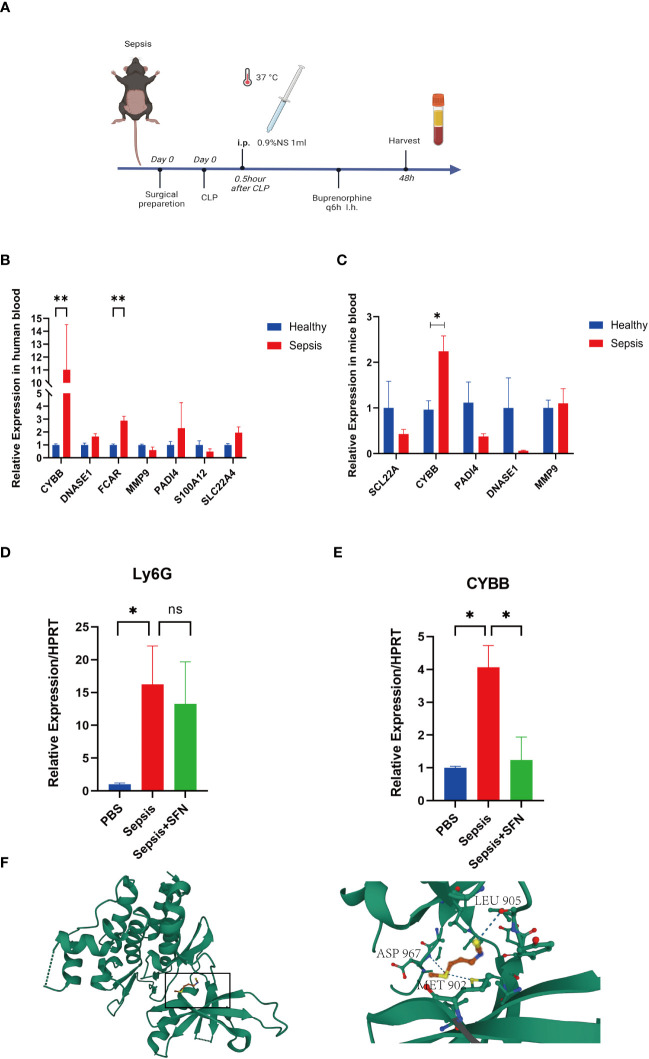
The validation of CYBB and FCAR in both humans and animals, and the validation of SFN in animals. **(A)** Sepsis modeling process. **(B)** Expression of NETs-related genes in peripheral blood of sepsis mice (n=6/group). **(C)** Expression of NETs-related genes in peripheral blood of septic patients (n=3/group). **(D)** The relative expression level of Ly6G RNA in mouse blood. **(E)** The relative expression level of CYBB RNA in mouse blood. (n=4/group, Data are presented as the means ± SE, *p<0.05, **p<0.01, and ns, not significant). **(F)** Prediction of the location of SFN’s action in the CYBB protein.

Bioinformatics analysis showed that *CYBB* and *FCAR* levels are related to the survival of patients with sepsis. Therefore, we collected 12 human whole blood samples(6 sepsis patiens and 6 healthy) and extracted RNA for analysis. *CYBB* and *FCAR* levels were significantly elevated in sepsis patients compared with the healthy group (**Figure 10B**).

In animal experiments, the mice were randomly divided into two groups by rolling dice, a sham group and a sepsis group, with 5 mice in each group. After 2 days, mice were sacrificed and their peripheral blood collected for RT-qPCR analysis. Notably, CYBB expression was increased in CLP group animals relative to controls (**Figures 10A, C**). According to the results of our drug prediction analysis, we identified three drugs predicted to target both FCAR and CYBB (**Figure S8**): 3,4,5,3,4’-pentachlorobiphenyl, antirheumatic agent, and sulforaphane(SFN), of which 3,4,5,3,4’-pentachlorobiphenyl is listed as a class 1 carcinogen by the World Health Organization, while antirheumatic drugs and SFN have potential to contribute to improvement of survival in patients with sepsis in the future. SFN is an isothiocyanate abundant in broccoli, and a common antioxidant that can both exert anti-inflammatory effects through the Nrf2/ARE pathway (23) and inhibit TNFα via RhoA/ROCK signaling (24). In a study of 45 people administered SFN for 14 days, 60% of patients with asthma showed improvement in symptoms and a significant reduction in airway resistance (25). In another study, macrophages were isolated from patients with chronic obstructive pulmonary disorder (n = 43) treated by SFN, and the results showed that macrophages increased the bacteria clearance rate by 40%–95%, and that inflammatory indicators were also reduced (26). Furthermore, SFN can improve the survival rate of mice with sepsis (27), and the drug has been tested in various clinical trials (28). In order to evaluate the specific location where SFN acts in the CYBB protein, we performed molecular docking analysis predictions. Using Autodock Vina v.1.2.2 (29), we obtained the binding pose and interactions between SFN and the CYBB protein. Our results indicate that SFN can bind to three sites in CYBB, namely MET902, LEU905, and ASP967, with a low binding energy of -3.632 kcal/mol (**Figure 10F**). Accordingly, we predict that SFN may be widely-used to treat patients with sepsis in the future.

**Figure 11:**
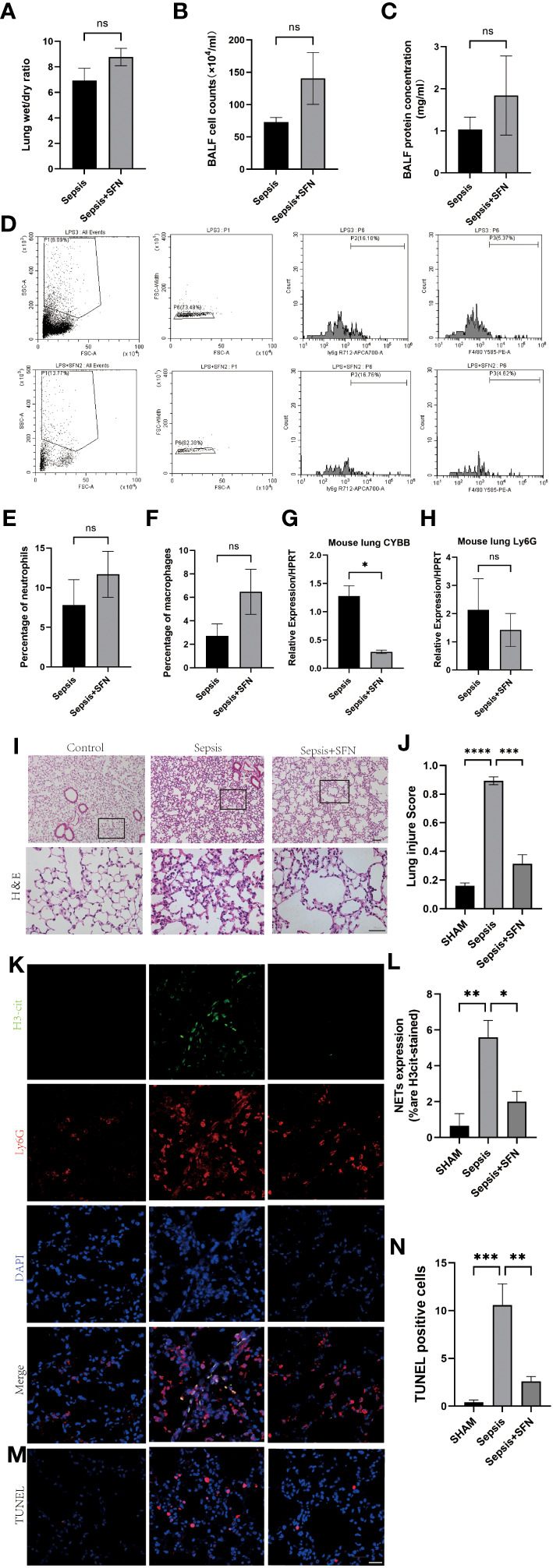
SFN reduce pulmonary NETs and CYBB, thereby repaired sepsis-induced lung injury. **(A)** Wet/dry ratio of mouse lung. **(B)** Cell count in bronchoalveolar lavage fluid (BALF) from mouse.(n=4/group). **(C)** Protein concentration in BALF from mouse. **(D)** Detection of Ly6G+ and F480+ cells in mouse BALF using flow cytometry. **(E)** Comparison of the number of Ly6G+ cells in mouse BALF using flow cytometry. **(F)** Comparison of the number of F480+ cells in mouse BALF using flow cytometry.(n=4/group). **(G)** Measurement of CYBB expression in mouse lung using qPCR. **(H)** Measurement of Ly6G expression in mouse lung using qPCR. **(I)** HE staining and local magnification of mouse lung (Upper scale bar=100 μm, The bottom scale bar=50um,n=4/group). **(J)** Comparison of lung injury score of mouse lung HE staining. **(K)** Immunofluorescence staining of mouse lung (H3cit in green, Ly6G in red, DAPI in blue, Scale bar=100 μm). **(L)** Comparison of NETs expression intensity in mouse lung immunofluorescence staining **(M)** TUNEL staining of mouse lung. **(N)** Comparison of the number of TUNEL-positive cells in three groups of mouse lung after TUNEL staining (Data are presented as the means ± SD. *p<0.05, **p<0.01, ***p<0.001, ****p<0.0001, and ns means not significant).

**Figure 12:**
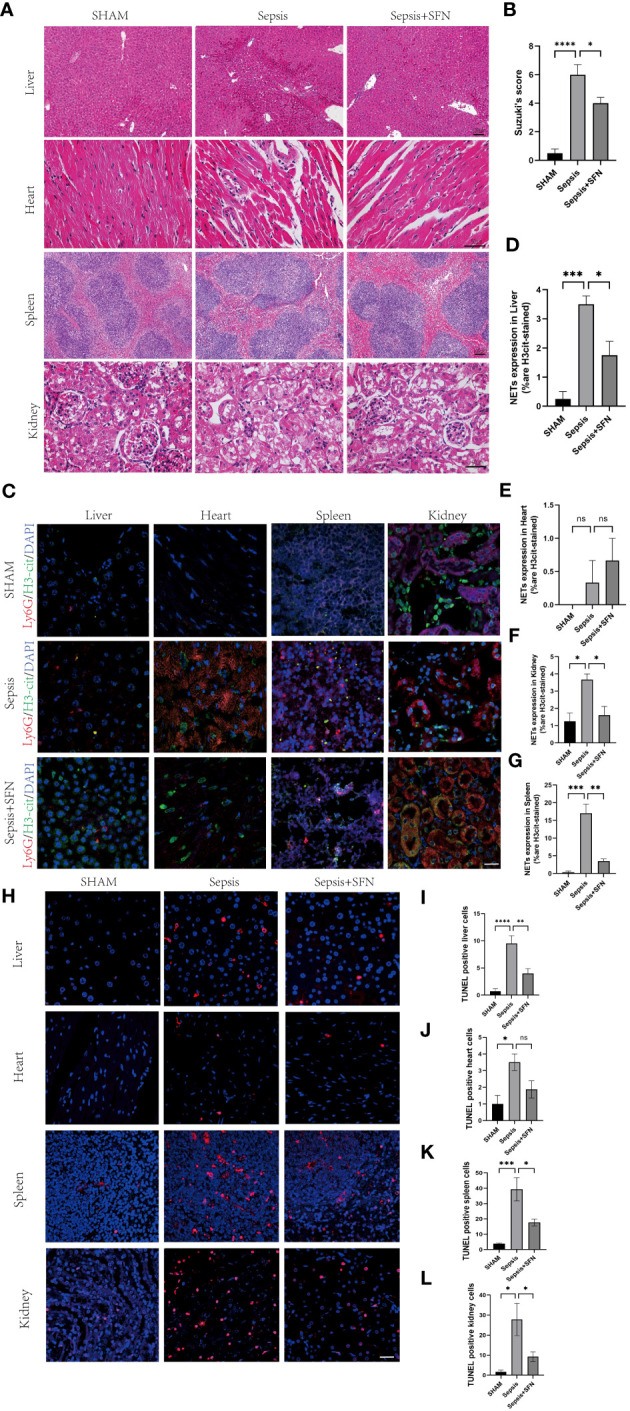
SFN alleviates sepsis-induced multi-organ damage by reducing the formation of NETs and decreasing apoptosis. **(A)** HE staining of mouse liver, heart, spleen, and kidney.(liver and spleen Scale bar=100 μm,heart and kidney Scale bar=50µm). **(B)** Evaluate the liver injury status of sepsis-induced liver histopathology (HE slides) using the Suzuki score.(n=4/group). **(C)** Immunofluorescence staining of mouse liver, heart, spleen, and kidney (Ly6G in red, H3-cit in green, DAPI in blue, Scale bar=100 μm, n=4/group). **(D)** Expression of NETs in liver cells. **(E)** Expression of NETs in heart cells. **(F)** Expression of NETs in spleen cells. **(G)** Expression of NETs in kidney cells. **(H)** TUNEL staining of mouse liver, heart, spleen, and kidney to observe apoptosis(n=4/group,Scale bar=100 μm). **(I)** Comparison of TUNEL-positive cells in mouse liver. **(J)** Comparison of TUNEL-positive cells in mouse heart. **(K)** Comparison of TUNEL-positive cells in mouse spleen. **(L)** Comparison of TUNEL-positive cells in mouse kidney (Data are presented as the means ± SD., *p<0.05, **p<0.01, ***p<0.001, ****p<0.0001, and ns means not significant).

We found that both *CYBB* and *FCAR* were expressed at high levels in normal human lung tissues (**Figure S7**), while *FCAR* was not expressed in murine tissues. Therefore, we administered SFN to further investigate use of this drug as a potential therapeutic strategy targeting CYBB signaling in mouse. The mice were divided into three groups, a sham group, a sepsis group, and a sepsis+SFN group, with 4 mice in each group.RT-qPCR showed that SFN suppressed *CYBB* expression *in vivo* (**Figure 10E**),

Meanwhile, there was no significant change in the expression of neutrophil Ly6G (**Figures 10D**, **11D, E**). Since CYBB is mainly expressed in the lungs in humans, we focused on the changes in lung injury caused by sepsis (**Figure 11**). and used American Thoracic Society work report method to assess lung injury (30). We then observed the pharmalogical effects on the liver, heart, spleen, and kidneys of septic mice (**Figure 12**).

Immunofluorescence(IF) analysis revealed that SFN significantly reduced sepsis-related lung injury by decreasing levels of NETs, but not wet/dry ratio of the lungs, balf cell count or balf protein concentration (**Figures 11A–C**).

To exclude the possibility that SFN influenced the number of neutrophils and macrophages, we next undertook FACS to count numbers of Ly6G^+^ cells and F4/80 ^+^ cells in BALF (**Figures 11D–F**). The results showed that there were no significant differences between the control and SFN groups, consistent with lung tissue Ly6G mRNA expression analysis (**Figures 11G, H**). In addition, we observed by IF that administration of SFN significantly contributed to alleviated sepsis-induced lung injury and apoptosis, accompanied by reduced (**Figures 11I–N**). Of note, our data confirmed that SFN also reduced NETs-related injury in organs such as liver,kidney, spleen but not heart (**Figures 12A–G**). These findings were consistent with the results of the TUNEL assay(**Figures 12 H–L**), further supporting the potential therapeutic value of SFN in treating sepsis-induced multi-organ damage.Thus, our data demonstrated the effects of CYBB inhibition on sepsis induced NETs-related multiple organs injury.

## 4 Discussion

The main purpose of this study was to identify key genes related to sepsis NETs using machine learning and verify them with *in vivo* experiments. Our data broaden understanding of the physiopathology and molecular mechanisms underlying sepsis, and provide new targets for sepsis treatment. We performed statistical analyses of two datasets (training and validation sets) using the limma package in R language to identify differentially expressed mRNAs. In the GSE65682 dataset, we detected 4931 up- and 4041 down-regulated genes. GO and KEGG analyses demonstrated that the DEGs were enriched for the mitochondrial matrix CC, consistent with previous research from our team on liver ischemia-reperfusion. Topological mapping showed that biological pathways including leukocyte cell-cell adhesion and lymphocyte differentiation were also enriched. Significantly associated genes and module membership were also assessed by WGCNA and three modules related to sepsis were screened. GO enrichment analysis of the genes in these three modules demonstrated that they were related to neutrophil activation involved in immune response, neutrophil-mediated immunity, ribonucleoprotein complex biogenesis, ATPase activity, and neutrophil degranulation, among other processes (**Figure S3D**). Based on these findings, combined with our previous research experience, we speculated that some key genes involved in NETs production during sepsis may play a role in the modules screened as important; therefore, we took the intersection of DEGs, key module genes, and NETs-related genes for functional analysis. Moreover, machine learning was applied to narrow down the eigengenes. Furthermore, to verify the selected signature genes GSEA, interaction, and PPI analyses were conducted to study the connection between NETs-associated genes and sepsis. Web-based tools were also used to predict miRNAs and TFs that regulate the seven screened genes related to sepsis NETs, and to predict drugs that can target the genes. Analysis of the GSE65682 dataset indicated that immune infiltration has an important role in sepsis, and that neutrophils were significantly upregulated in sepsis. Additionally, the GSE145227 dataset was selected for use in verification analysis and to explore the clinical significance of the seven genes identified as related to sepsis NETs (*S100A12*, *SLC22A4*, *FCAR*, *CYBB*, *PADI4*, *DNASE1*, *MMP9*), which all had diagnostic significance. In particular, *CYBB* and *FCAR* were significantly associated with survival in patients with sepsis, and both genes were validated using animal experiments and analysis of clinical specimens.

Several previous studies have linked FCAR and CYBB to sepsis. FCAR encodes a transmembrane glycoprotein receptor in the Fc region of IgA, which is highly expressed in neutrophils. Wehrli et al. (31) found that FCAR can stimulate changes in cells, including cytoplasmic vacuolization, mitochondrial swelling, and nuclear pyknosis. Further, FCAR can regulate neutrophil activity and induce various forms of neutrophil death, consistent with the results of our research. As shown in **Figure 7**, research on NETs to date has focused on IgG, but IgA, as the second largest immune protein, also plays a key role in inflammatory immunity, similar to FCγR, which can lead to recruitment of the downstream tyrosine kinase, SYK, which causes inflammatory damage (32). Different from FCAR, CYBB, also known as NOX2, has been extensively studied in sepsis.Giusy Tiseo et al. discovered that levels of soluble NOX2-derived peptides were significantly elevated in sepsis patients and septic shock patients compared to healthy controls, and a stair-like increase was observed between the two patient groups. The deceased group showed a tendency towards higher levels of soluble NOX2-derived peptides compared to the survival group, which was consistent with the results of our study (33). Additionally, Joseph LC et al. found that NOX2 increases oxidative stress, leading to mitochondrial dysfunction and septic myocardial injury (34). Further studies found that CYBB can lead to an increase in NETs by stimulating ROS (35). Previous research in our laboratory demonstrated that changes in mitochondrial permeability transition pore (mPTP) can affect mitochondrial ROS and thus NETs generation. Further, we used bioinformatics analysis to predict that elevated CYBB will lead to increased mPTP, thereby increasing NETs. Husain et al. (36) found that inhibiting CYBB could not only reduce neutrophil immunosuppression in critically ill patients with sepsis, but also reduce sepsis bacterial infection and organ damage, similar to the findings of our study. Inhibition of CYBB can also inhibit microglial activation and improve cognitive impairment after sepsis (37), as well as promoting sepsis myocardial injury through the ERK1/2-TNFα pathway (38).

The formation of NETs is currently believed to occur through three pathways. In the first pathway, induced by Staphylococcus aureus, neutrophil nuclear membranes rupture, and vesicles form, encapsulating DNA that is transferred to the cell membrane. These vesicles then fuse with the cell membrane, releasing the enclosed DNA into the extracellular space, thus forming NETs. This process typically takes 30 to 60 minutes (39). In this pathway, the neutrophil’s nucleus and cell membrane remain intact, preserving the cell’s activity and phagocytic function. Consequently, it is also known as active NETs (40). The second pathway involves neutrophil nuclear membrane degradation mediated by phorbol myristate acetate (PMA). In this process, the chromatin within the neutrophil condenses, leading to eventual cell lysis after approximately 3 to 4 hours (41). This pathway is characterized by activated neutrophils undergoing chromatin decondensation and subsequent cell death, and is thus referred to as suicidal NETs.The third pathway is a ROS-dependent mechanism in which neutrophils release mitochondrial DNA to form NETs. This process is associated with changes in ROS-induced NADPH (42). While PAD4 is an important target and CI-Amidine has demonstrated efficacy in reducing NETs and improving sepsis, its role in inhibiting NET formation in the mitochondrial NETs pathway is limited (9). As described in **Figure 7**, CYBB can increase ROS and mtROS levels through NADPH, thereby influencing the generation of mitochondrial pathway NETs. This can further enhance therapeutic efficacy and reduce adverse outcomes of NETs-associated inflammatory diseases.

Our study had some limitations. First, a larger patient cohort was warranted. Although we verified the gene expression findings in mice, in an effort to overcome the inherent limitations of bioinformatics technology, we must recruit more patients in future studies. Second, our data on NETs-related genes are based on bioinformatics predictions, which have a high false positive rate; therefore, more experimental data are needed to validate our findings. Finally, the infection period of sepsis was not explored in the study as this information was not included in the GEO data, and NETs behave differently during different periods; hence, more detailed clinical information should be included in future analyses.

Together, we illustrated by machine learning that CYBB and FCAR were significantly associated with sepsis-related mortality. Our study demonstrated the therapeutic potential of targeting the two NETs-related genes in treatment of sepsis.

## Data availability statement

The original contributions presented in the study are included in the article/**Supplementary Materials**, further inquiries can be directed to the corresponding author/s.

## Ethics statement

The studies involving humans were approved by Medical Ethics Committee of Sun Yat-sen University Third Affiliated Hospital, Guangzhou. The studies were conducted in accordance with the local legislation and institutional requirements. The participants provided their written informed consent to participate in this study. The animal study was approved by Institutional animal care and use committee,jennio biotech co.ltd. The study was conducted in accordance with the local legislation and institutional requirements.

## Author contributions

GHY: Data curation, Formal Analysis, Investigation, Methodology, Project administration, Software, Validation, Visualization, Writing – original draft, Writing – review & editing. XGZ: Formal Analysis, Investigation, Methodology, Writing – review & editing. JRL: Data curation, Investigation, Methodology, Supervision, Writing – review & editing. KY: Investigation, Supervision, Writing – review & editing. XMY: Data curation, Investigation, Supervision, Writing – review & editing. HTC: Funding acquisition, Resources, Supervision, Writing – review & editing. XXW: Conceptualization, Funding acquisition, Writing – review & editing. YNH: Resources, Supervision, Writing – review & editing. XYY: Methodology, Writing – review & editing. YGL: Project administration, Writing – review & editing. ZPL: Validation, Writing – review & editing. YFH: Supervision, Writing – review & editing. MMF: Supervision, Resources, Writing – review & editing. YLA: Supervision, Validation, Writing – review & editing. TYL: Funding acquisition, Supervision, Writing – review & editing. HJL: Funding acquisition, Resources, Supervision, Visualization, Writing – review & editing, Conceptualization, Validation. XS: Funding acquisition, Resources, Supervision, Visualization, Formal Analysis, Investigation, Methodology, Writing – review & editing. HMY: Conceptualization, Funding acquisition, Project administration, Resources, Supervision, Visualization, Writing – review & editing.
