# Supplementary material for: Machine learning-based identification of CYBB and FCAR as potential neutrophil extracellular trap-related treatment targets in sepsis
Source: Front Immunol. 2023 Oct 13;14:1253833. doi: 10.3389/fimmu.2023.1253833 (PMC10613076; doi:10.3389/fimmu.2023.1253833)
Supplement: Supplementary file 1 [file DataSheet_1.pdf]

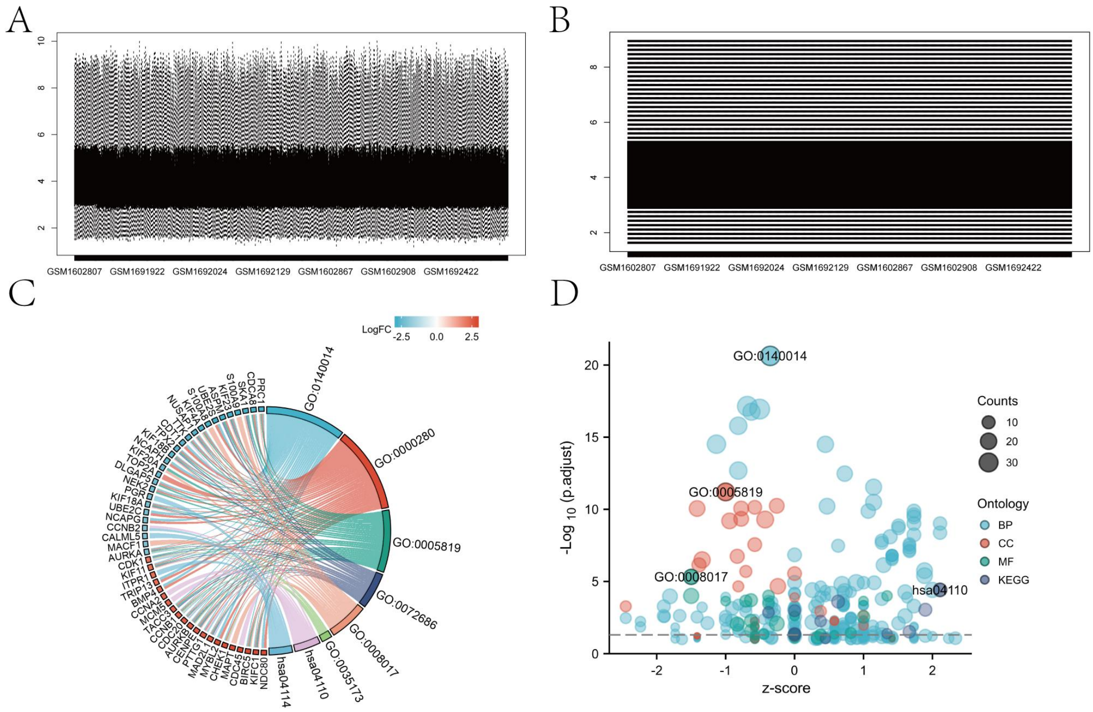

Supply fig1

A Data before normalization

B Normalized data

C DEGs GO KEGG string diagram

D DEGs bubble chart

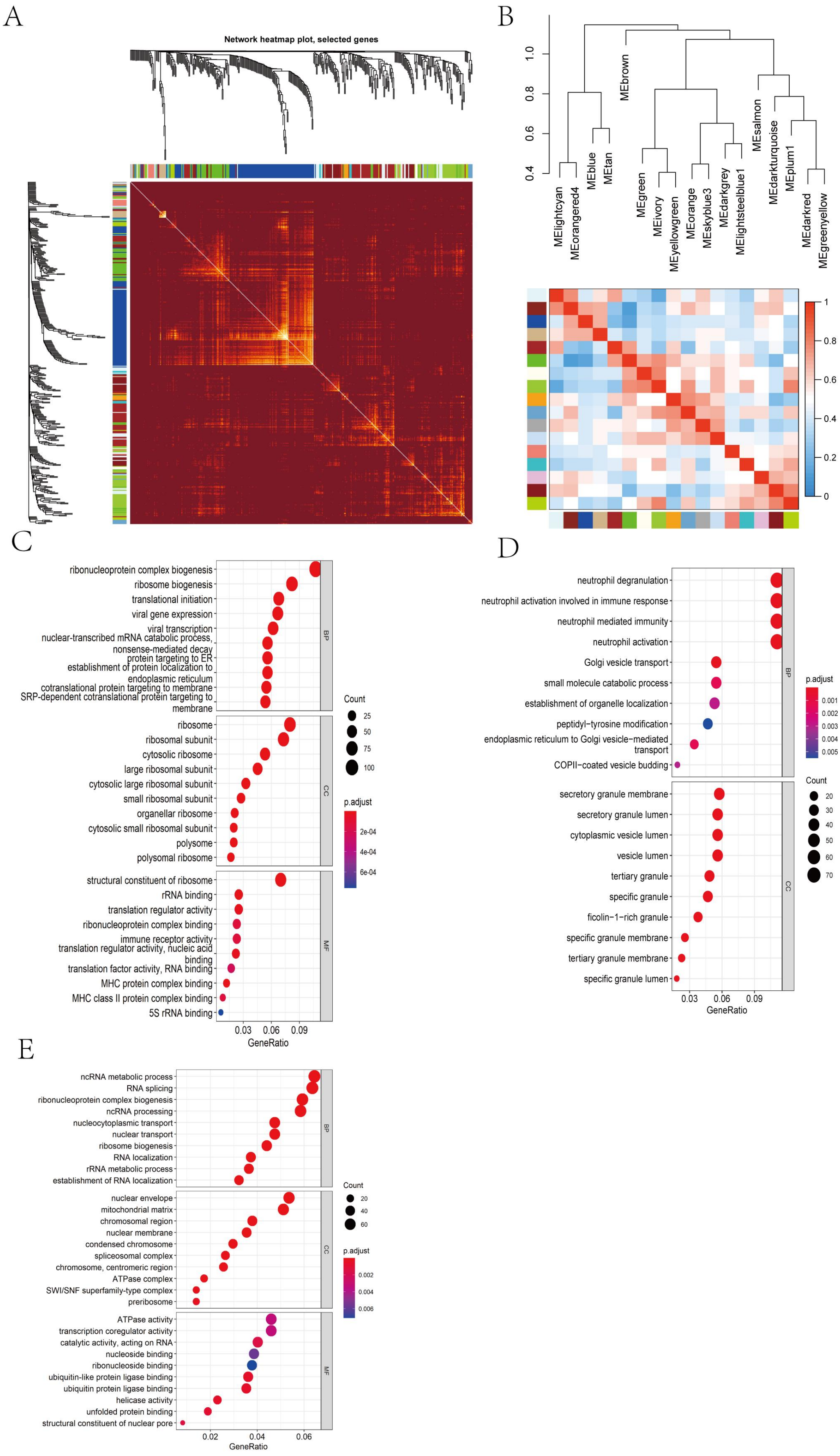
A
Network heatmap plot, selected genes
B
MElightcyan
MEorangered4
MEblue
MEtan
MEbrown
MEgreen
MEivory
MEyellowgreen
MEorange
MEskyblue3
MEdarkgrey
MElightsteelblue1
MEsalmon
MEdarkturquoise
MEplum1
MEdarkred
MEgreenyellow
C
ribonucleoprotein complex biogenesis
ribosome biogenesis
translational initiation
viral gene expression
viral transcription
nuclear-transcribed mRNA catabolic process, nonsense-mediated decay
protein targeting to ER
establishment of protein localization to endoplasmic reticulum
cotranslational protein targeting to membrane
SRP-dependent cotranslational protein targeting to membrane
ribosome
ribosomal subunit
cytosolic ribosome
large ribosomal subunit
cytosolic large ribosomal subunit
small ribosomal subunit
organellar ribosome
cytosolic small ribosomal subunit
polysome
polysomal ribosome
structural constituent of ribosome
rRNA binding
translation regulator activity
ribonucleoprotein complex binding
immune receptor activity
translation regulator activity, nucleic acid binding
translation factor activity, RNA binding
MHC protein complex binding
MHC class II protein complex binding
5S rRNA binding
BP
CC
MF
Count
p.adjust
GeneRatio
D
neutrophil degranulation
neutrophil activation involved in immune response
neutrophil mediated immunity
neutrophil activation
Golgi vesicle transport
small molecule catabolic process
establishment of organelle localization
peptidyl−tyrosine modification
endoplasmic reticulum to Golgi vesicle−mediated transport
COPII−coated vesicle budding
secretory granule membrane
secretory granule lumen
cytoplasmic vesicle lumen
vesicle lumen
tertiary granule
specific granule
ficolin−1−rich granule
specific granule membrane
tertiary granule membrane
specific granule lumen
BP
CC
p.adjust
Count
GeneRatio
E
ncRNA metabolic process
RNA splicing
ribonucleoprotein complex biogenesis
ncRNA processing
nucleocytoplasmic transport
nuclear transport
ribosome biogenesis
RNA localization
rRNA metabolic process
establishment of RNA localization
nuclear envelope
mitochondrial matrix
chromosomal region
nuclear membrane
condensed chromosome
spliceosomal complex
chromosome, centromeric region
ATPase complex
SWI/SNF superfamily-type complex
preribosome
ATPase activity
transcription coregulator activity
catalytic activity, acting on RNA
nucleoside binding
ribonucleoside binding
ubiquitin-like protein ligase binding
ubiquitin protein ligase binding
helicase activity
unfolded protein binding
structural constituent of nuclear pore
BP
CC
MF
Count
p.adjust
GeneRatio

Supply fig2

A Clustering dendrogram of module feature genes.

B Collinear heat map of module feature genes.(Red color indicates a high correlation, blue color indicates opposite results.)

C yellow green module gene GO analysis

D light cyan module gene GO analysis

E green module gene and GO analysis

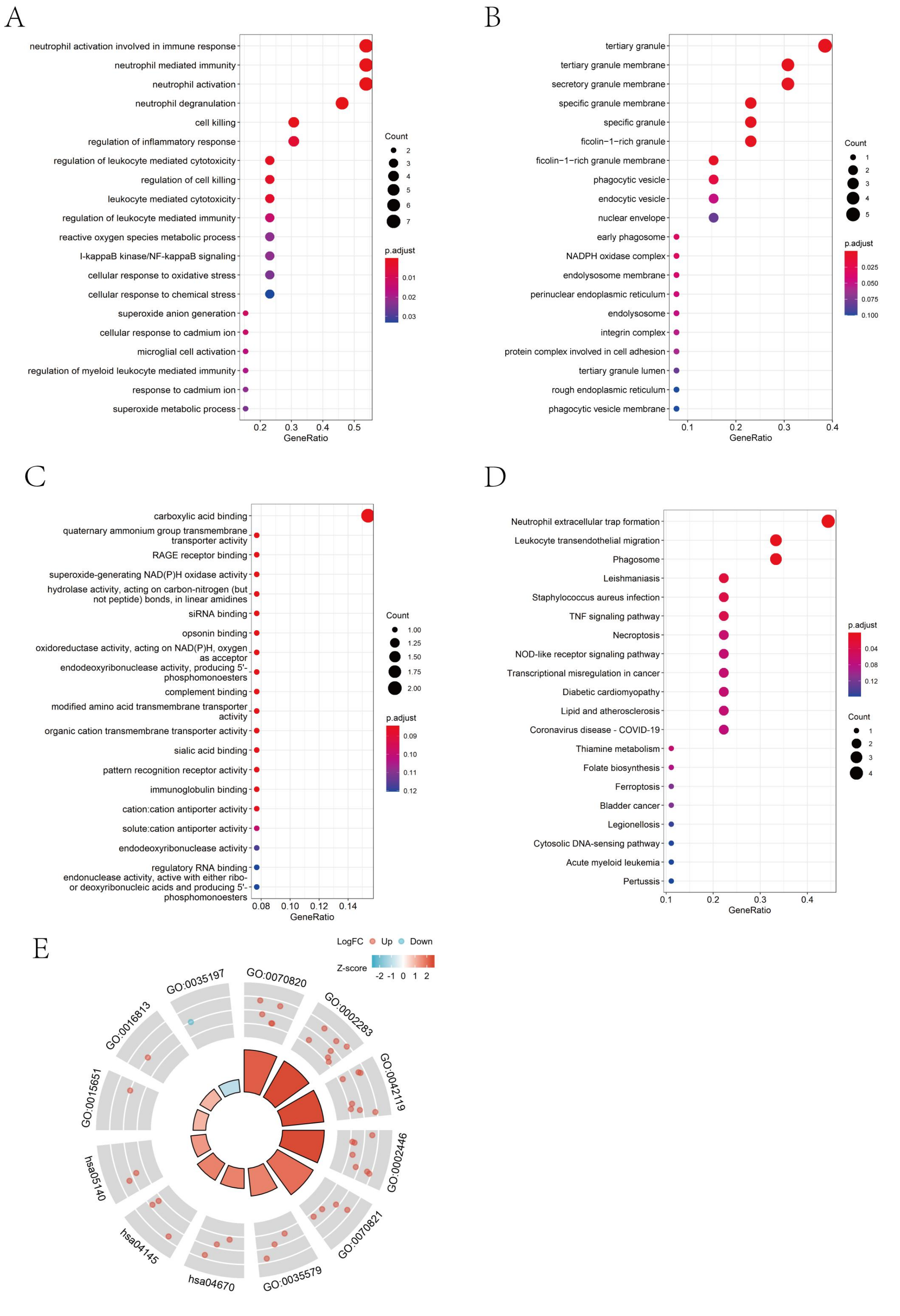

Supply fig3

A BP of 13 NETs-related genes

B CC of 13 NETs-related genes

C MF of 13 NETs-related genes

D KEGG of 13 NETs-related genes

E GO-KEGG circle plot of 13 NETs-related genes.

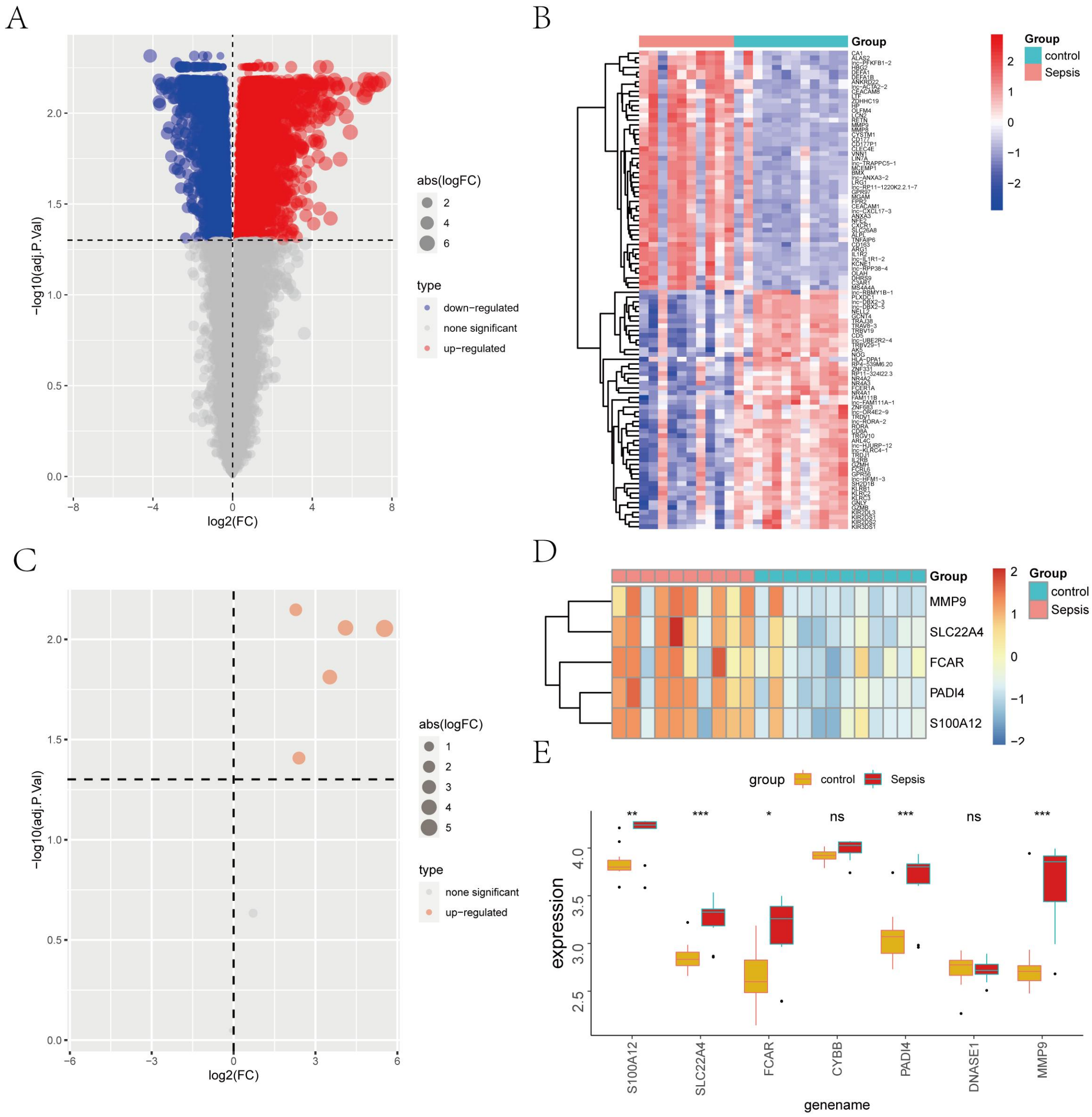

Fig4 Select GSE145227 as the authentication set

A-B difference analysis volcano map, heat map

C 7 genes were shown separately in the volcano map in GSE145227

D 7 genes are shown separately in the heat map in GSE145227

E Box plot of differential expression of 7 genes in GSE145227

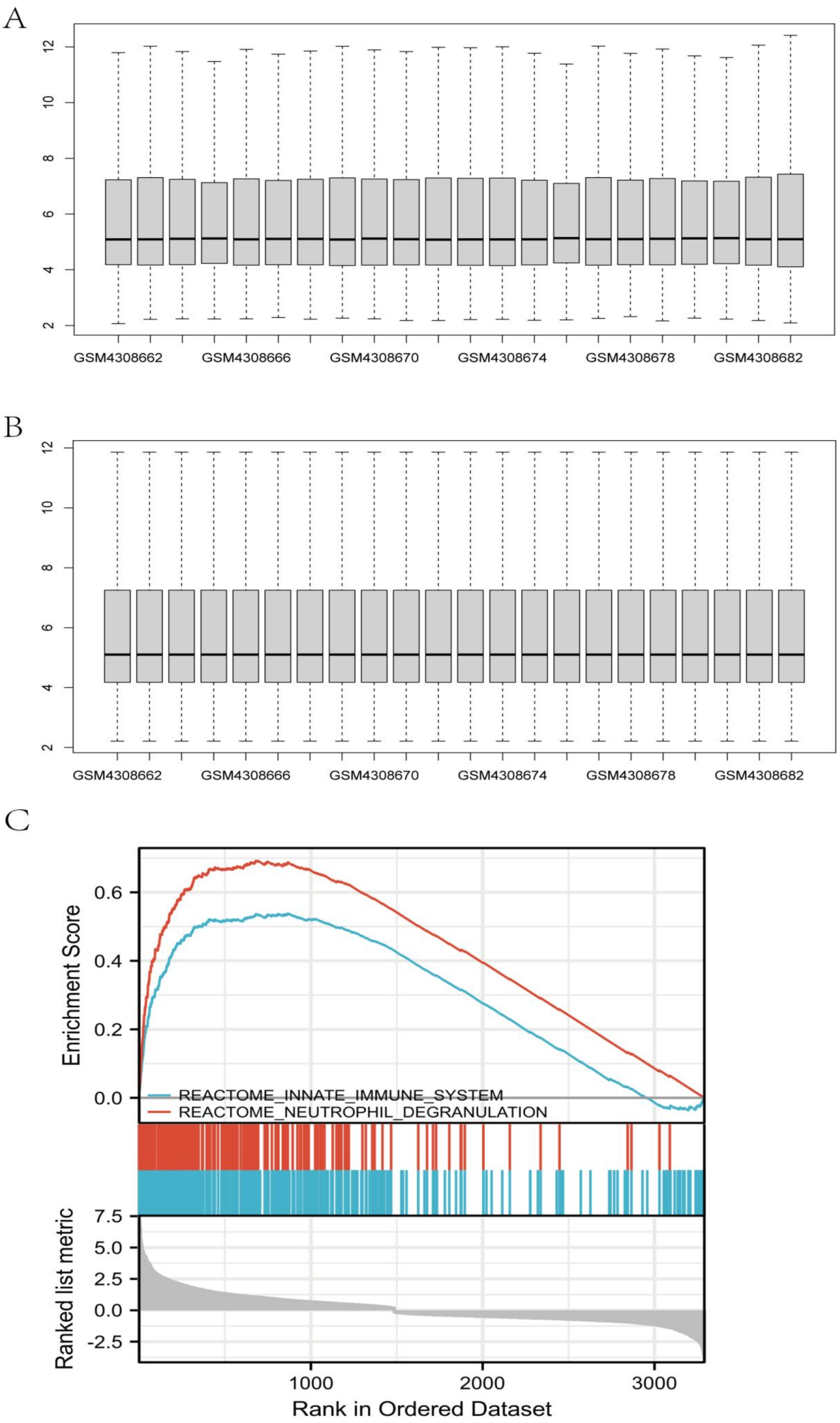
A
GSM4308662
GSM4308666
GSM4308670
GSM4308674
GSM4308678
GSM4308682
B
GSM4308662
GSM4308666
GSM4308670
GSM4308674
GSM4308678
GSM4308682
C
Enrichment Score
REACTOME_INNATE_IMMUNE_SYSTEM
REACTOME_NEUTROPHIL_DEGRANULATION
Ranked list metric
Rank in Ordered Dataset

Supply fig5

A GSE145227 dataset before normalization

B GSE145227 after normalized

C GSEA analysis

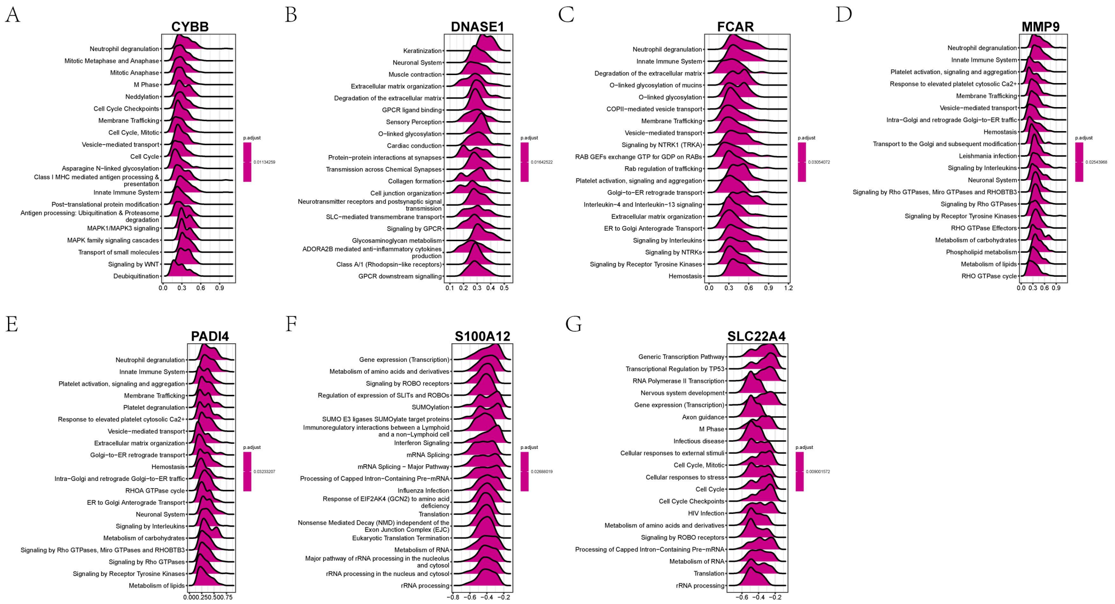

Supply fig6

Correlation analysis of 7 genes with all genes was performed using data from GSE65682, and based on the results of correlation analysis, Reactome-based GSEA analysis (R clusterprofiler package) was performed for single genes. The results of each of the top 20 of each of the seven genes were shown.

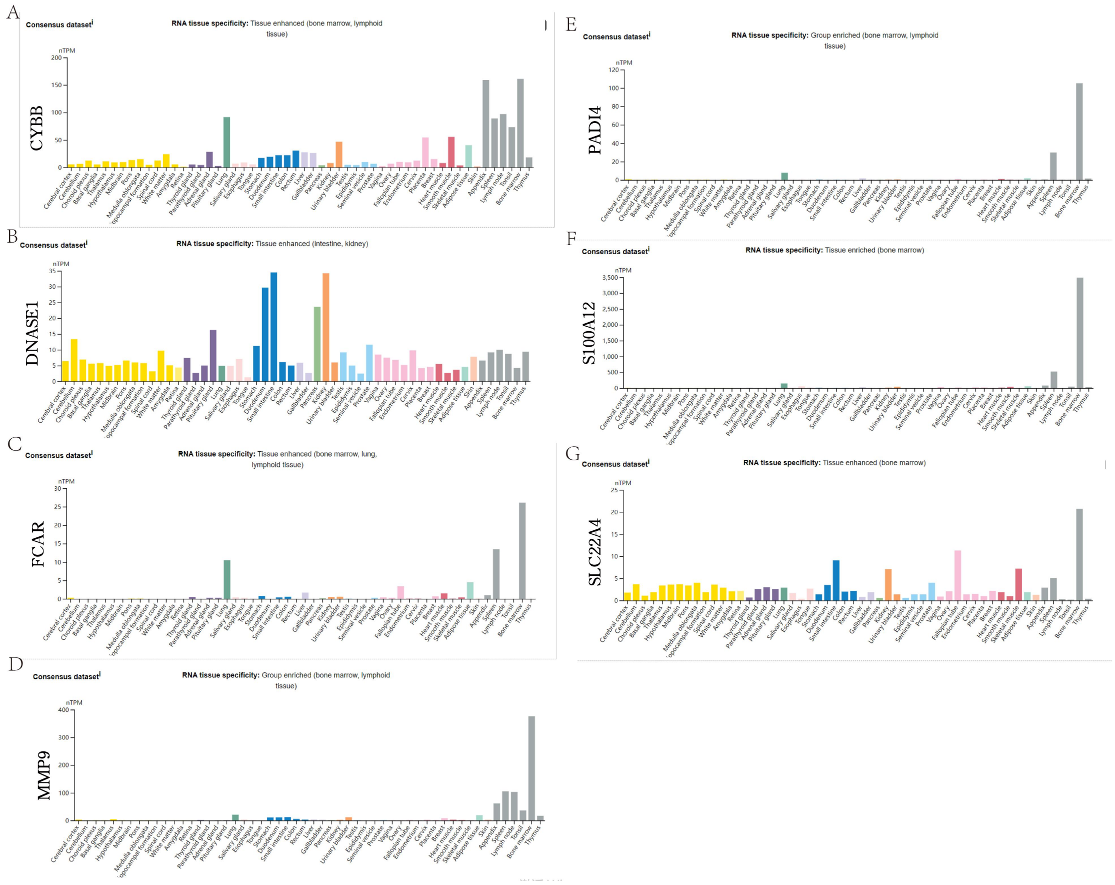

Supply fig7

Expression of 7 NETs-associated genes in normal tissues.（https://www.proteinatlas.org/）

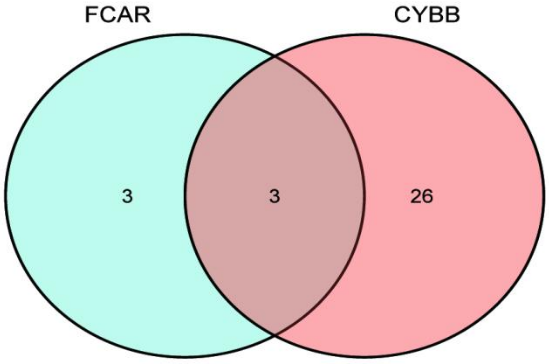

Supply fig8

Drug prediction Wayne diagram
